# Surgical treatment of autosomal recessive bestrophinopathy with angle-closure glaucoma: vitreous liquefaction as the key to correcting postoperative malignant glaucoma—three case reports

**DOI:** 10.3389/fmed.2025.1560475

**Published:** 2025-10-31

**Authors:** Zhonghua Sun, Cuijuan Liu, Wei Liu, Miaomiao Zhang, Shanshan Ren, Lei Gao, Zhen Ji

**Affiliations:** ^1^Department of Ophthalmology, Jinan Second People’s Hospital, Jinan, China; ^2^Nova Southeastern University College of Optometry, Miami, FL, United States

**Keywords:** autosomal recessive bestrophinopathy, angle-closure glaucoma, malignant glaucoma, vitreous humor, compound trabeculectomy

## Abstract

**Purpose:**

This study aimed to evaluate the surgical treatment of autosomal recessive bestrophinopathy (ARB) combined with angle-closure glaucoma (ACG) through a retrospective case series.

**Methods:**

The treatment of three patients with ACG secondary to ARB was reviewed. The patients were admitted to the Department of Ophthalmology of Jinan Second People’s Hospital from April 2023 to January 2024. Their conditions, treatments, and outcomes were extracted from the medical records and analyzed.

**Results:**

The patients were 48, 48, and 35 years old at the time of surgery. All had bilateral ARB and underwent surgery in the eye more severely affected by ACG. Topical eye drops failed to control the intraocular pressure (IOP), which measured 27, 28, and 47 mmHg before the surgery. The affected eyes also exhibited a shorter axial length (AL) and shallower anterior chamber depth (ACD). The ALs of the surgical eyes measured 22.73 mm, 21.52 mm, and 20.96 mm, while the ACDs were 2.51 mm, 1.97 mm, and 2.19 mm, respectively. After receiving trabeculectomy, they all immediately developed malignant glaucoma, which could not be resolved by conservative treatment. Following a second surgery, which importantly included an anterior vitrectomy and posterior capsulotomy, the IOP was normal, the ACD was satisfactory, and visual function was preserved.

**Conclusion:**

For ACG/ARB patients, the risk of developing malignant glaucoma after glaucoma surgery is very high. Surgical intervention, such as anterior vitrectomy, is needed to increase vitreous fluidity, eliminate vitreous block, assist the formation of the anterior chamber, and stabilize the IOP to save the patient’s vision. Long-term, close follow-up is essential due to the risk of recurrence in the operated eye and occurrence in the non-operated eyes.

## Introduction

Autosomal recessive bestrophinopathy (ARB) is a rare retinal degenerative disorder (1). It is caused by biallelic mutations in the BEST1 gene, which are typically characterized by homozygous or compound heterozygous mutations. These result in a greater degree of gene function loss and more extensive lesions. Genetic testing is required to make the diagnosis. The primary lesion of ARB is located in the retinal pigment epithelium (RPE) which might cause the maldevelopment of both the anterior and posterior segments of the eyes characterized by more pronounced crowding of the anterior ocular segment including short axial length (AL), narrow angle, shallow anterior chamber (2), choroidal thickening (3, 4), as well as an increased risk of developing ACG compared to other variants (5, 6).

Current treatment options are primarily symptomatic and supportive, encompassing the correction of macular edema and the monitoring of glaucoma. Surgical interventions are crucial to managing the complications associated with ARB and preventing vision loss. For example, trabeculectomy can be used to reduce the intraocular pressure (IOP) and thus protect the optic nerve (7), and surgeries such as goniosynechialysis and laser peripheral iridotomy (LPI) can prevent further closure of the anterior chamber angle (8). However, the probability of malignant glaucoma and refractory shallow anterior chamber after these surgeries is very high (9). In complex situations, a combination of lens extraction, trabeculectomy, and anterior vitrectomy may be necessary. Experience sharing is particularly important for physicians to be educated and prepared to deal with ARB, as no unified clinical treatment guideline has been established.

Unfortunately, there are few reports on the occurrence of malignant glaucoma or refractory shallow anterior chambers after the surgical treatment of ARB patients, and there are even fewer reports on the treatment of such complications (10–12). In this article, we share our experiences by reviewing the treatment of three ARB patients with angle-closure glaucoma (ACG), who developed malignant glaucoma immediately after the initial surgery. We evaluated the relationship between postoperative complications (malignant glaucoma and refractory shallow anterior chamber) and the clinical characteristics of the patients.

## Methods

This study was approved by the Ethics Committee of Jinan Second People’s Hospital (Approval No. JNEYE20240641) and complied with the Declaration of Helsinki. Written informed consent was obtained from all patients or their families.

Routine examinations included best-corrected visual acuity (BCVA), Goldmann applanation tonometry, slit lamp microscopy with goniometry, and fundus examination with autofluorescence imaging. In addition, the ocular biometry profile was assessed using an IOLMaster700 instrument (Carl Zeiss, Germany), and an average value of 10 measurements was taken. The visual field was measured using a Humphrey 750 visual field analyzer (Carl Zeiss, Germany) operating the 30-2 SITA Fast program. During the examination, patients with refractive errors were corrected with glasses, and the mean deviation (MD) was recorded. To determine the angle structure and quantify angle closure, the anterior segment structure was scanned using an SW3200L ultrasound biomicroscope (Tianjin Suowei Electronic Technology Co., Ltd., Tianjin, China) in a room with 60–70 Lux illumination. The retina and choroid were examined under natural pupil conditions by OCT using a Cirrus HD-OCT 4000 instrument (Carl Zeiss, Germany). For color fundus photography, the posterior pole of the fundus was photographed under natural pupil conditions using an Optos Daytona P200T ultra-widefield retinal imaging device (Optos, United Kingdom).

They were diagnosed with ARB based on typical fundus changes in the non-invasive retinal imaging examination, and the diagnosis was verified by their genetic testing results. Specifically, multifocal, subretinal, yellow-white deposits in the posterior pole were detected by color fundus photography. These lesions were mostly around the optic disc, near the retinal vascular arch, or in the macular area. Fundus autofluorescence (FAF) imaging with confocal scanning laser ophthalmoscopy revealed that vitelliform lesions exhibited hyperautofluorescence, distributed predominantly in the macular area, posterior pole, and equatorial regions. However, the fovea typically demonstrated hypoautofluorescence. The OCT showed strongly reflective deposits at the subretinal RPE level, which corresponded to the multifocal yellow-white deposits in the fundus (13).

The diagnosis with ACG is based on the following: (1) history of repeated elevation of IOP (>21 mmHg), with or without symptoms, (2) narrow or closed anterior angle observed in ultrasound biomicroscopy or gonioscope, and (3) glaucomatous optic disc changes and visual field damage.

Malignant glaucoma diagnosis in postoperative cases was confirmed through comprehensive evaluation of key clinical indicators: (1) observed shallowing or complete collapse of the anterior chamber morphology, (2) documented elevation of IOP measurements (>21 mmHg), and (3) definitive exclusion of potential choroidal pathologies, including effusion manifestations and suprachoroidal hemorrhagic events.

In the event of malignant glaucoma, immediate conservative treatment, including mydriasis, intraocular pressure-lowering therapy, and anti-inflammatory therapy, was initiated. If conservative treatment proves ineffective, prompt surgical intervention with anterior vitrectomy combined with posterior capsulotomy is performed. The management procedures were as follows: An infusion cannula was inserted through the limbal side port to maintain anterior chamber stability. A 23-gauge sclerotomy was created 3.5 mm posterior to the corneal limbus, followed by anterior vitrectomy and central posterior capsulotomy. The diameter of the posterior capsule incision was recommended to be approximately 3–4 mm. The cutting rate was set between 500 and 1,000 cuts per minute. Sufficient anterior vitreous was excised until significant anterior chamber deepening was observed and intraocular pressure returned to normal levels.

## Results

### Patient information

Table 1 summarizes the basic information of the three patients, including one 48-year-old man and two women aged 48 and 35. Patient 3 had bilateral LPI at another hospital 7 years ago. None of the patients had a family history of eye diseases, and using eye drops failed to control the IOP.

**Table 1:** Patient information.

Patient	Sex (M/F)	Age (year)	Medical history	ARB medications	Intraocular pressure (mmHg)	
					Left	Right
1	M	48	Hypertension	Brinzolamide/timolol eye drops, brimonidine tartrate eye drops	27^a^	19
2	F	48	None	Brinzolamide/timolol eye drops, brimonidine tartrate eye drops	28^a^	17
3	F	35	Thyroidectomy	Carteolol hydrochloride eye drops, brinzolamide eye drops	21	47^a^

a The eye receiving surgery. For all three patients, both eyes were affected by ARB.

### Exam findings

#### Routine exams and biometry

**Figure 1:**
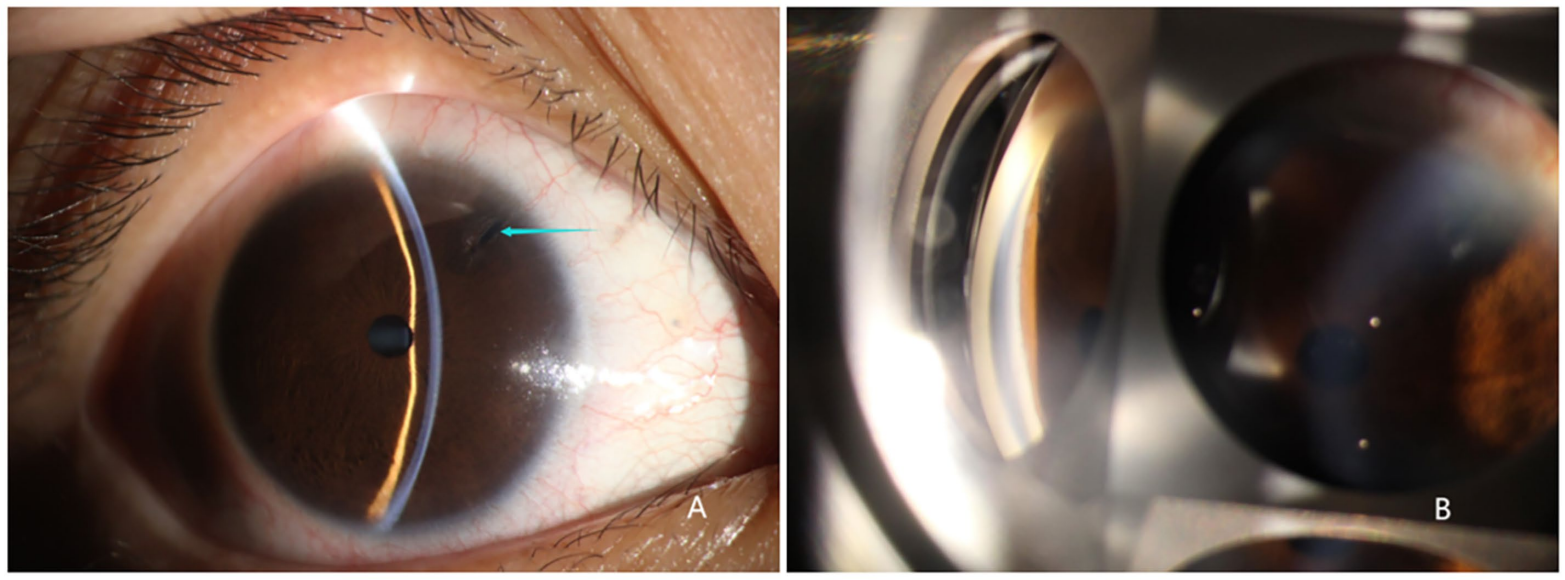
Slit lamp examination of the right eye of patient 3. **(A)** Transparent cornea, shallow anterior chamber, bulging iris, good patency of the incision from the prior iridotomy, round pupil with ~2 mm diameter post miosis, transparent lens in place. **(B)** Grade IV and gonioscopically narrow according to the Scheie classification. Angle structures were completely invisible, and the angle was closed.

The slit lamp examination of all three patients showed that the cornea was transparent, the anterior chamber was shallow, the iris had a clear texture, the lens was in place, and there was no inflammation or posterior synechiae. For patient 3, the peripheral iridotomy incision was patent (Figure 1A). For all three patients, the eye scheduled for surgery was classified, according to the Scheie system, as grade IV and gonioscopically narrow. None had a visible anterior chamber angle structure, and the angle was always closed (Figure 1B). Their ALs were 22.73, 21.52, and 20.96 mm, and the ACDs were 2.51, 1.97, and 2.19 mm, respectively. The BCVA (LogMAR) before surgery was 0.4, 1.0, and 0.3, respectively, and the preoperative IOP was 27, 28, and 47 mmHg, respectively (Table 2).

**Table 2:** Conditions of the eye before the initial surgery.

Patient	BCVA (LogMAR)	IOP (mmHg)	AL (mm)	ACD (mm)	LT (mm)
1	0.4	27	22.73	2.51	4.50
2	1.0	28	21.52	1.97	4.02
3	0.3	47	20.96	2.19	4.77

BCVA, best-corrected visual acuity; IOP, intraocular pressure; AL, axial length; ACD, anterior chamber depth; LT, lens thickness.

#### Ultrasound biomicroscopy

**Figure 2:**
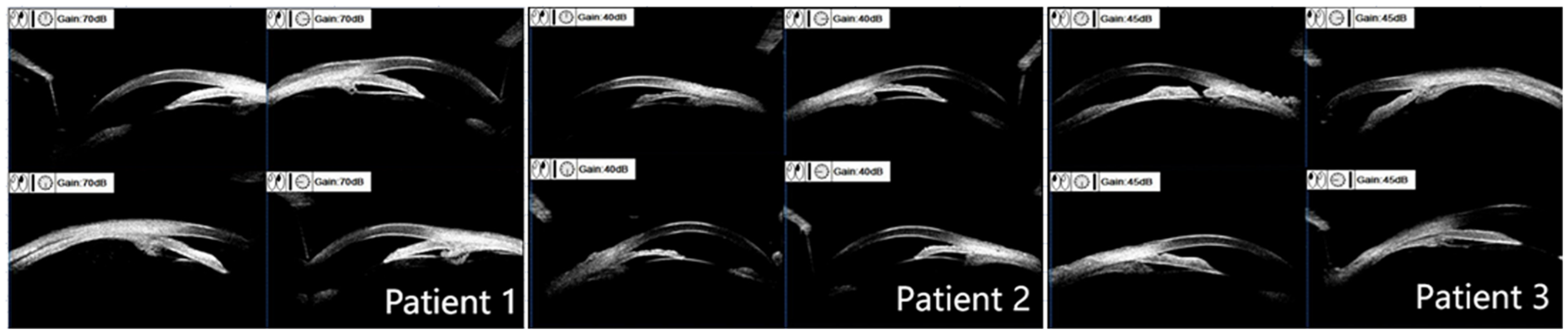
Ultrasound biomicroscope examination results. Patients 1 and 2: mild iris bulging, mild anterior displacement of the ciliary body, open temporal quadrant with an extremely narrow gap, all other quadrants closed, normal lens position. Patient 3: anterior displacement of the ciliary body in all four quadrants, scleral protrusion blocked by the iris root, chamber angle completely closed, patent peripheral iridotomy incision at the 1 o’clock position.

All three patients presented with iris bulging, anterior displacement of the ciliary body, and obstruction of the scleral spur at the iris root. The lens was in a normal position. For patients 1 and 2, one quadrant of the chamber angle was open with an extremely narrow gap, and the rest was all closed. For patient 3, complete angle closure was observed in all four quadrants, and the peripheral iridotomy incision was patent at the 1 o’clock position (Figure 2).

#### Optical coherence tomography

**Figure 3:**
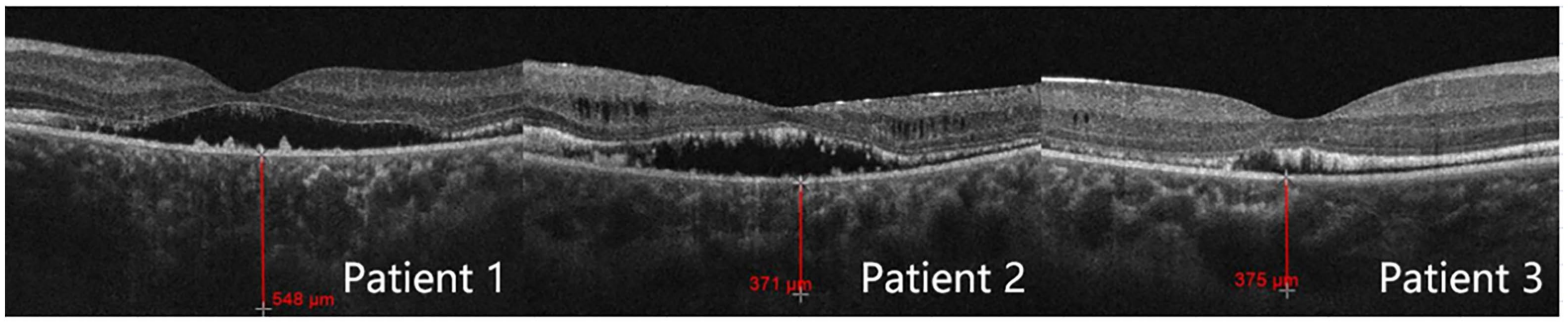
Subfoveal choroidal thickness measured by optical coherence tomography.

For all three patients, the OCT optic disc examinations revealed an increase in the cup/disc ratio and a decrease in the mean retinal nerve fiber layer (RNFL) thickness. The OCT macula examinations revealed that the central foveal thickness (CFT) increased, and the subfoveal choroidal thickness (SFCT) increased (Table 3). There was serous detachment of the foveal neuroretina, and the choroid thickened (Figure 3).

**Table 3:** Test results of optical coherence tomography.

Patient	Mean RNFL thickness (μm)	CFT (μm)	SFCT (μm)	C/D
1	61	258	548	0.85
2	58	208	371	0.71
3	90	220	375	0.78

RNFL, retinal nerve fiber layer; CFT, central foveal thickness; SFCT, subfoveal choroidal thickness; C/D, cup/disc ratio.

#### Color fundus photography

**Figure 4:**
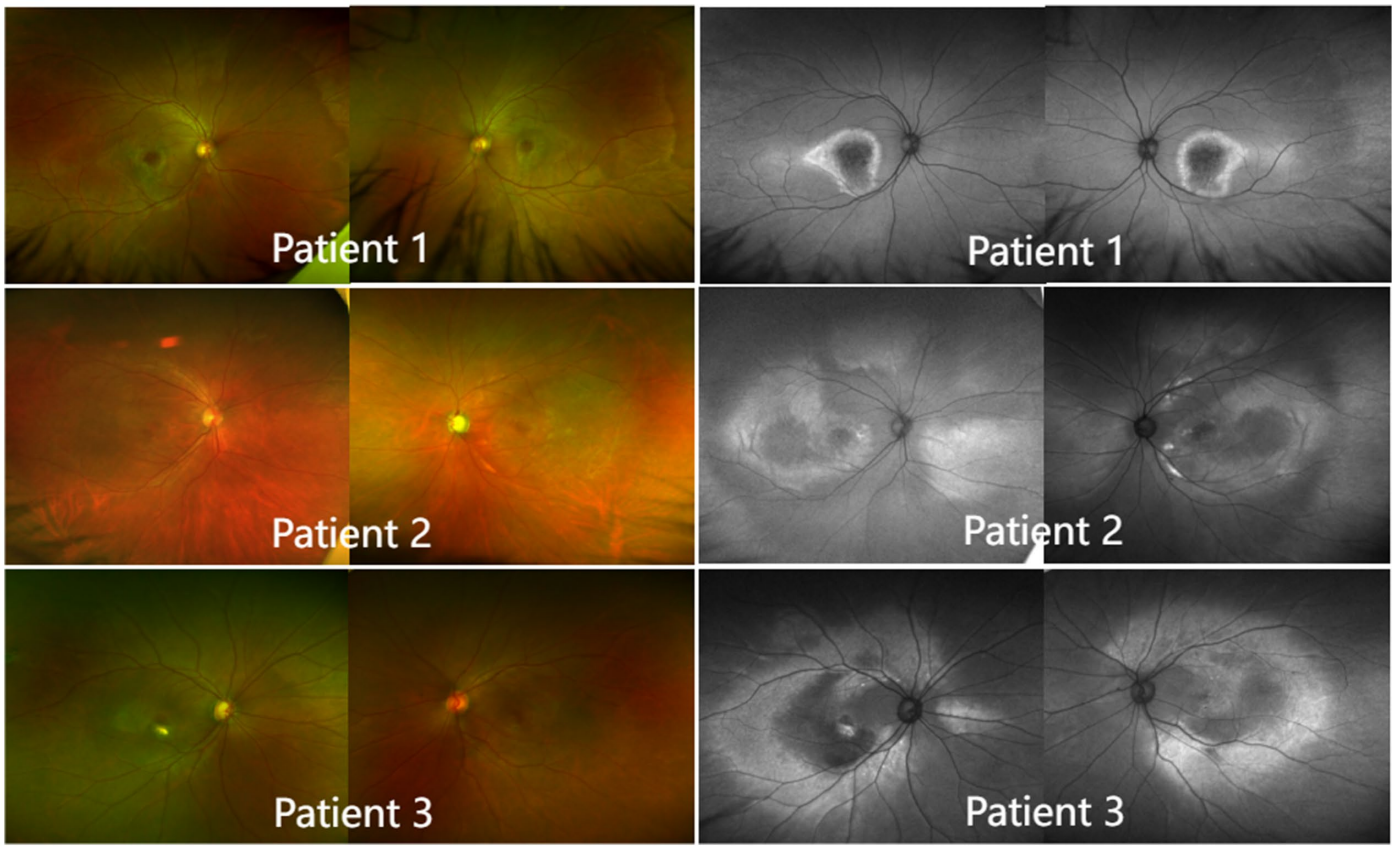
Color fundus photography and autofluorescence imaging (left). In all patients, multifocal subretinal yellow-white materials, which varied in number and size and appeared in the form of spots or clusters, were deposited in the posterior pole of both eyes. Autofluorescence imaging (right). The fovea had weak fluorescence. With its strong fluorescence, the posterior pole could be clearly distinguished from the peripheral fundus, which had normal fluorescence. At the boundary between the posterior pole and the peripheral fundus, there were multiple hyperfluorescent spots arranged in a nearly circular pattern.

For all patients, multifocal subretinal yellow-white materials, which varied in number and size and appeared in the form of spots or clusters, were deposited in the posterior pole of both eyes. The autofluorescence images showed widespread and uneven enhancement or reduction. The fovea had weak fluorescence. With its strong fluorescence, the posterior pole could be clearly distinguished from the peripheral fundus that had normal fluorescence. On the boundary between the posterior pole and the peripheral fundus, there were multiple hyperfluorescent spots arranged in a nearly circular pattern (Figure 4).

#### Genetic testing

The type of genetic detection was whole exome. All patients had compound heterozygous mutations. Patient 1 detected two heterozygous variations in the BEST1 gene: *c.481 + 1G > A/c.256G > T(p.A86S)*, patient 2 detected two variations in BEST1 gene: *c.584C > T(p.A195V)/c.653G > A(p.R218H)*, and patient 3 detected two compound heterozygous variations in the BEST1 gene: *c.584C > T(p.A195V)/c.763C > T(p.R255W)*.

#### Case summary

Patient 1 (48-year-old man) presented to our hospital with blurred vision in his left eye for 1 month. His diagnosis was later confirmed by genetic testing. Despite treatment with a triple topical antiglaucoma medication regimen, the IOP remained at 27 mmHg. Consequently, the patient underwent a compound trabeculectomy in the left eye. On the first day after the surgery, malignant glaucoma occurred with shallow ACD (grade II), and the IOP was 23 mmHg. Due to the clear crystalline lens status, the patient refused secondary surgical intervention and was treated conservatively, including reducing posterior chamber pressure, anti-inflammatory steroids, and mydriasis. The shallow ACD persisted (grades I to II). After 4 months of recurring symptoms and worsening medication compliance, the patient finally consented to a combined procedure that included lens extraction, IOL implantation, anterior vitrectomy, and posterior capsulotomy.

Patient 2 (48-year-old woman) presented to our hospital with a 2-year history of blurred vision in her left eye. Comprehensive ophthalmic evaluation and genetic testing established the diagnosis of ARB. Despite treatment with triple topical antiglaucoma medications, the IOP was controlled at 28 mmHg. Due to concurrent lens opacity, the patient initially underwent combined phacoemulsification with intraocular lens implantation and trabeculectomy in the left eye. On the first day after the surgery, malignant glaucoma occurred with shallow ACD (grade II), and the IOP was 25 mmHg. After being treated conservatively, including reducing posterior chamber pressure, anti-inflammatory steroids, and mydriasis, the shallow ACD persisted (grades I to II). On the 18th day after the initial surgery, she received an anterior vitrectomy and posterior capsulotomy.

**Figure 5:**
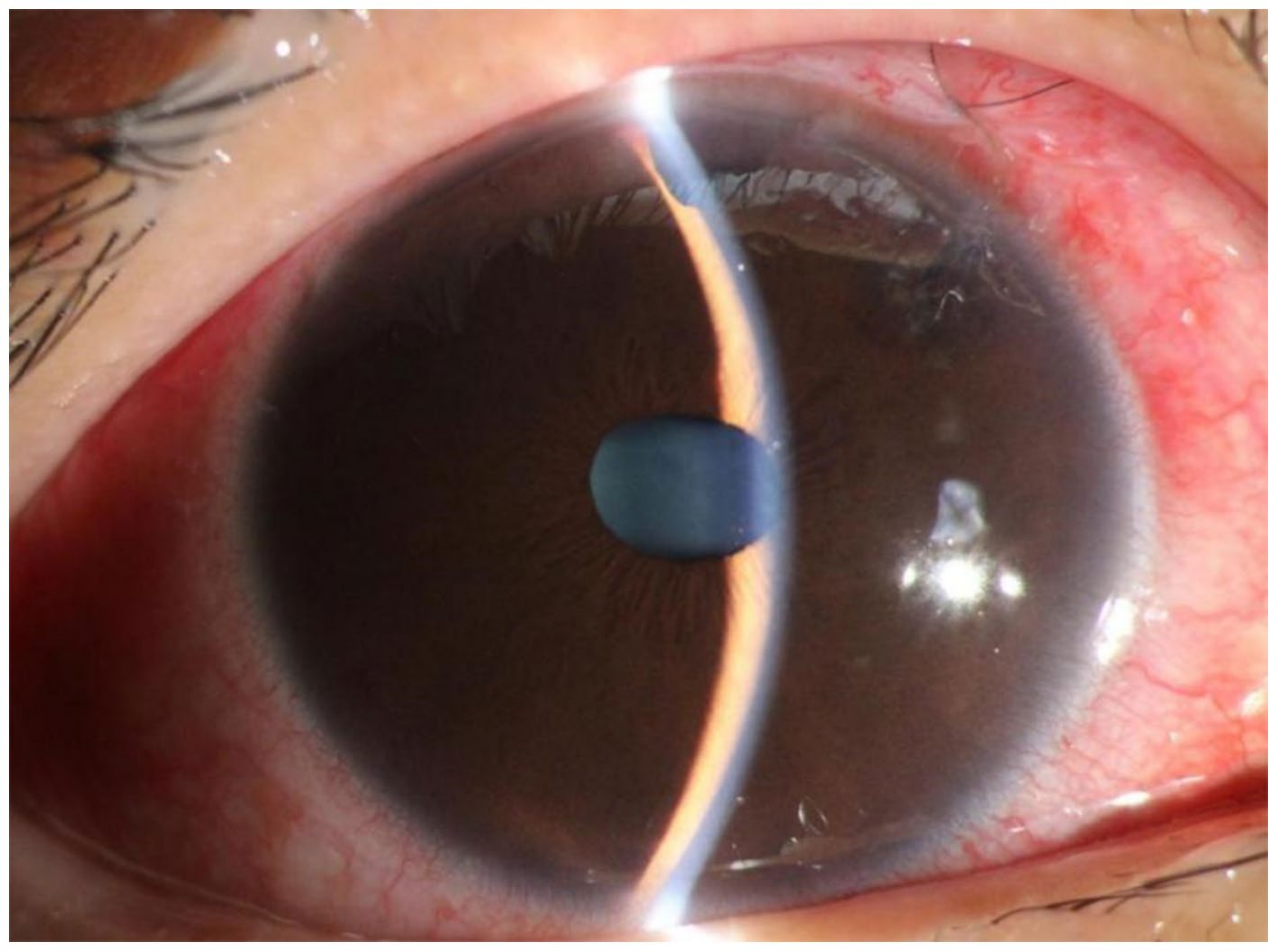
Slit lamp examination of patient 3 on the first day after compound trabeculectomy. The findings included conjunctival hyperemia, diffuse bulging of the filtering bleb above the conjunctiva, good incision apposition, sutures in place, transparent cornea, disappearance of the anterior chamber, iris bulging, patent superior iris incision, and transparent lens.

Patient 3 (35-year-old woman) presented with a 7-year history of bilateral ACG previously diagnosed at an external hospital, having undergone bilateral Laser Peripheral Iridotomy (LPI). Despite treatment with two antiglaucoma medications, the IOP in her right eye remained elevated at 47 mmHg. She was diagnosed with ACG and ARB, and then underwent compound trabeculectomy in the right eye. On the first day after the surgery, malignant glaucoma occurred with shallow ACD (grade III), and the IOP was 35 mmHg. The conservative treatment was ineffective; the anterior chamber did not deepen, and secondary corneal fogging occurred (Figure 5). To avoid further exacerbation, on day 2 after the initial surgery, she received anterior vitrectomy and posterior capsulotomy, along with lens extraction and IOL implantation.

**Figure 6:**
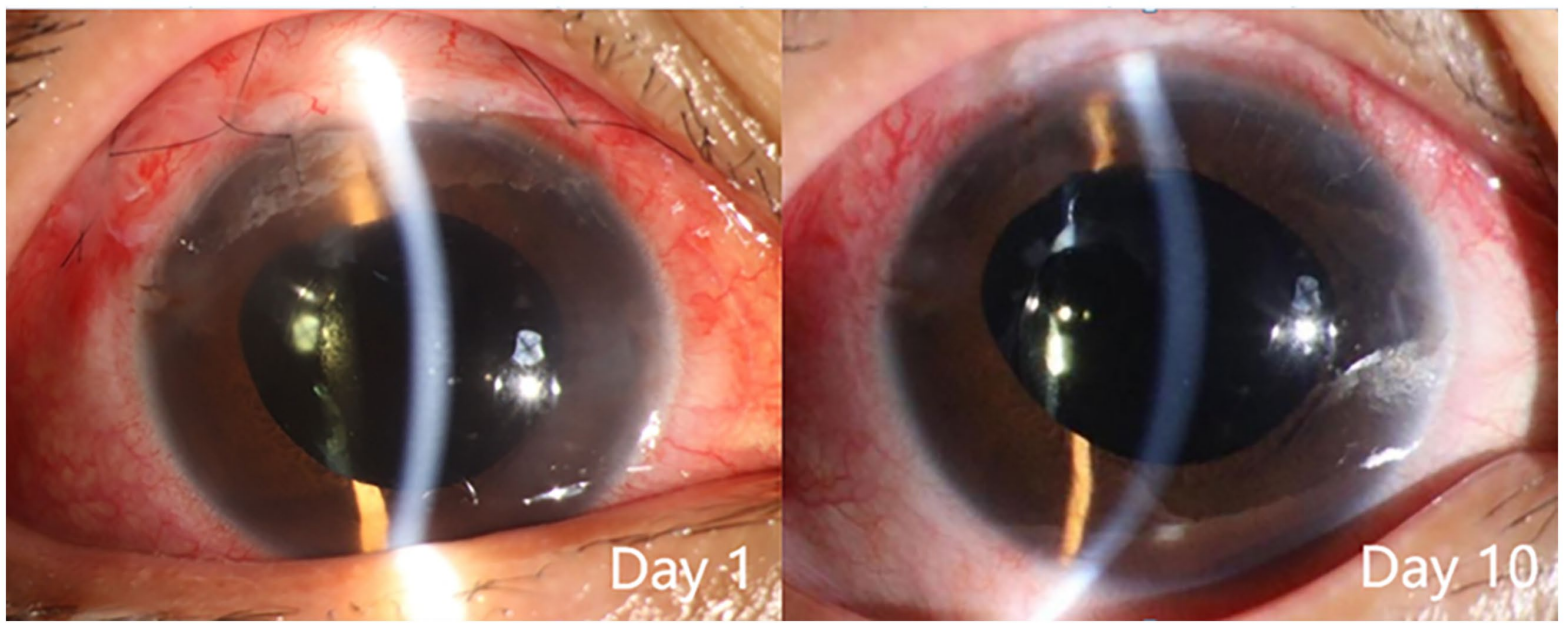
Slit lamp examination of patient 3 after the second surgery. The findings included: day 1, conjunctival hyperemia, good alignment of the conjunctival incision, sutures in place, diffuse elevation of the upper filtering bleb, transparent cornea, normal anterior chamber depth, moderate pupil dilation due to medication, transparent intraocular lens in place, and incision of the central posterior capsule; day 10, reduced conjunctival hyperemia, sutures removed, diffuse elevation of the upper filtering bleb, transparent cornea, normal anterior chamber depth, moderate pupil dilation due to medication, transparent intraocular lens in place, and transparent area of central posterior capsulotomy.

For all three patients, the anterior chamber was formed on the first day after the second surgery. During the next 3 months, the IOP remained stable, and the anterior chamber was well formed (Figure 6). The follow-up at 3 months after the surgery confirmed the efficacy of their treatment, as the visual function was preserved, the IOP was normal, and the ACD was good (Table 4).

**Table 4:** Key data of the operated eye at 3 months after anterior vitrectomy.

Patient	BCVA (LogMAR)	IOP (mmHg)	ACD (mm)
1	0.7	13	3.54
2	0.7	10	3.41
3	0.3	14	3.23

BCVA, best-corrected visual acuity; IOP, intraocular pressure; ACD, anterior chamber depth.

The non-surgical eyes of these three patients were treated with topical anti-glaucoma medications, and their IOP was effectively controlled within the normal range. Considering that their current IOP was well-managed, surgical intervention was not currently warranted. However, close follow-up was required to monitor disease progression.

## Discussion

The three patients in this study were aged 48, 48, and 35, respectively. Patient 3 had the shortest AL and the earliest onset. Lin et al. (14) found that 19.3% of ACG patients who are less than 40 years old have ARB, but 44.8% of these patients are misdiagnosed as primary ACG, uveitis, or macular edema. As in the fundus examination without mydriasis, the subretinal vitelliform lesions can be easily overlooked due to the closed angle. We thus recommend that physicians apply a comprehensive evaluation when diagnosing young ACG patients and bear in mind the possibility of ARB, especially when a dilated eye examination is not feasible.

Genetic testing is crucial for a definitive diagnosis. The three patients had compound heterozygous mutations. According to the “ACMG (American College of Medical Genetics and Genomics) Standards and Guidelines for the Classification of Genetic Variants” (15), the variants “c.481 + 1G > A,” “c.584C > T(p.A195V),” and “c.763C > T(p.R255W)” are classified as “pathogenic variants.” The variants “c.256G > T(p.A86S)” and “c.653G > A(p.R218H)” are classified as a “likely pathogenic variant.” Literature reports indicate that these variants have been detected in multiple patients with ARB presenting with similar symptoms. Included are several homozygous cases or compound heterozygous relationships with known (likely) pathogenic variants, such as c.830C > T, c.73C > T, c.598C > T, c.1066C > T, and c.38G > A (2, 16, 17).

With anterior segment abnormal development, it is particularly difficult to treat the ACG associated with ARB. LPI and cataract extraction may not reduce IOP satisfactorily (5, 8, 18). In this study, patient 3 experienced failure in IOP control following bilateral LPI. Trabeculectomy is generally the first-line surgical treatment for ACG in the Chinese population, especially for patients with >180° peripheral anterior synechiae and no significant lens opacities (19–21). However, for ACG/ARB patients, trabeculectomy alone tends to cause postsurgical malignant glaucoma (21, 22). Clear lens extraction is a rational surgical option when malignant glaucoma occurs (23), but most ACG/ARB patients are young and often reluctant to accept clear lens extraction initially. Hence, it is imperative to thoroughly communicate with the patients about their condition and inform them of the likelihood of a second surgery. In this study, both patients 1 and 3 had healthy, clear lenses, and they refused to undergo clear lens extraction along with trabeculectomy. Unfortunately, a refractory shallow anterior chamber occurred in both patients on the first day after the initial surgery.

Moreover, some studies have suggested that even combining lens removal with trabeculectomy may not suffice in preventing postoperative refractory shallow anterior chamber (24). Of the three patients described in this study, the lens thickness was within the normal range in all three cases. Patient 2 underwent lens removal along with trabeculectomy, but still developed malignant glaucoma after the initial surgery. We thus speculate that, for ACG/ARB patients, the angle closure mechanism may not be due to pupillary blockage upon lens enlargement. Instead, the culprit may be the pressure from the posterior segment, which pushes forward the iris diaphragm and thus reduces the ACD (8). Quigley et al. (25) pointed out that the thickening choroid pushes the molded vitreous body forward, creating a high-pressure environment and causing vitreous conduction. The choroidal thickness of patients 1, 2, and 3 was 548, 371, and 375 μm, respectively. The study by Huo et al. (26) revealed that the mean subfoveal choroidal thickness in healthy adults was 288.43 ± 74.60 μm for the 31–40 years group and 278.05 ± 72.87 μm for the 41–50 years group. Notably, the choroidal thickness in our three patients was significantly greater than that of age-matched healthy adults in the corresponding age groups. Since ARB patients are usually young, the vitreous has a high degree of integrity and a low degree of liquefaction, and choroidal thickening passes greater pressure onto the anterior chamber. Once the anterior vitreous contacts the ciliary body and the lens, the effective diffusion area decreases, and the reflux of the aqueous humor occurs. Surgeries such as trabeculectomy create additional outflow channels and can further increase the pressure gradient between the anterior and posterior segments. The lens-iris diaphragm moves further forward and causes angle closure. At this point, the aqueous humor does not have anywhere else to go other than entering the vitreous cavity. Therefore, for patients, especially younger patients with a thicker choroid, glaucoma drainage surgery should be selected with extra caution since the probability of postoperative malignant glaucoma is very high.

For all three patients described in this study, a second surgery to correct the malignant glaucoma may require a combination of anterior vitrectomy, lens extraction, and posterior capsulotomy may be necessary. Following crystalline lens removal, the reduced volume of the intraocular lens and capsular bag increases the ciliary-lenticular space. Posterior capsulotomy disrupts the relative integrity of the lens capsule. Anterior vitrectomy disrupts the integrity of the anterior hyaloid membrane and vitreous block (27). Combined with the communicating effects of peripheral iridotomy and posterior capsulotomy, this approach establishes an antero-posterior communication (28). For most malignant glaucoma patients, this combined surgical approach demonstrates satisfactory therapeutic outcomes (29). Studies have reported a surgical success rate of 64% in such cases (30).

Shi et al. contended that the abnormalities in the ciliary body-zonules-crystalline lens-hyaloid-anterior vitreous (CZLHV) complex play a key role in the development of malignant glaucoma (31). The surgical procedure of irido-zonulo-hyaloid-vitrectomy (IZHV), which directly targets the CZLHV complex, has become a commonly used method for the treatment of malignant glaucoma. However, when only IZHV was performed, the postoperative recurrence rate remained as high as 66%. Some scholars have postulated that this recurrence might be attributed to insufficient anterior vitreous volume reduction, potentially leading to recurrent obstruction of the aqueous outflow pathways by residual vitreous humor (32). Parames et al. (9) reported that all five cases of BEST1 gene-associated glaucoma patients developed malignant glaucoma following filtration surgery, which were effectively managed through combined IZHV along with pars plana vitrectomy (PPV). Therefore, we propose that regardless of the surgical approach adopted, particular attention should be paid to achieving sufficient excision of the anterior vitreous to prevent residual vitreous displacement caused by posterior pressure due to elevated intravitreal pressure and recurrent malignant glaucoma. Tian et al. (33) applied transscleral cyclophotocoagulation (TSCPC) to treat ARB patients who developed malignant glaucoma after the combined surgery of phacoemulsification, IOL implantation, and goniosynechialysis (34). These findings further substantiate the crucial role of improving anterior vitreous liquefaction with higher motility in the therapeutic strategy for malignant glaucoma.

In summary, we reviewed the treatment of three ARB patients with ACG, with a focus on the management of postoperative malignant glaucoma. We recommend that physicians carefully select the suitable treatment for young ACG patients, who are more likely to have secondary ACG, and perform thorough fundus examinations to rule out undiagnosed genetic diseases. For ACG/ARB patients receiving trabeculectomy, with or without concomitant lens removal, there is a very high risk of postoperative malignant glaucoma, and improving the fluidity of the anterior vitreous via combined anterior vitrectomy, diode laser TSCPC, or IZHV may be necessary. We believe that comprehensive evaluations and thorough communication are key for ophthalmologists when treating young ACG patients without cataracts. Simultaneously, long-term, close follow-up is also necessary to monitor the condition of both eyes in patients, to timely detect changes in IOP, and to ensure the visual function of patients over an extended period. The limitation of this study lies in the small sample size, and we anticipate collecting more cases in future studies to provide clinically relevant insights.

## Data Availability

The original contributions presented in the study are included in the article/supplementary material, further inquiries can be directed to the corresponding author.
